# GABAergic‐astrocyte signaling: A refinement of inhibitory brain networks

**DOI:** 10.1002/glia.23644

**Published:** 2019-05-30

**Authors:** Sara Mederos, Gertrudis Perea

**Affiliations:** ^1^ Department of Functional and Systems Neurobiology Instituto Cajal, CSIC Madrid Spain

**Keywords:** astrocyte, excitation/inhibition balance, GABAergic interneurons, gliotransmission, synaptic plasticity

## Abstract

Interneurons play a critical role in precise control of network operation. Indeed, higher brain capabilities such as working memory, cognitive flexibility, attention, or social interaction rely on the action of GABAergic interneurons. Evidence from excitatory neurons and synapses has revealed astrocytes as integral elements of synaptic transmission. However, GABAergic interneurons can also engage astrocyte signaling; therefore, it is tempting to speculate about different scenarios where, based on particular interneuron cell type, GABAergic‐astrocyte interplay would be involved in diverse outcomes of brain function. In this review, we will highlight current data supporting the existence of dynamic GABAergic‐astrocyte communication and its impact on the inhibitory‐regulated brain responses, bringing new perspectives on the ways astrocytes might contribute to efficient neuronal coding.

## 1 INTRODUCTION

Complex cognitive functions depend on a proper balance between the excitatory and inhibitory synapses belonging to different types of excitatory and inhibitory neurons, which define structurally and functionally distinct subnetworks. Therefore, the concept of excitation/inhibition (E/I) balance denotes the relative contributions of excitatory and inhibitory synaptic inputs, and its significance is reinforced by the fact that synaptic imbalances (synaptopathies) seem to underlie different brain disorders including epilepsy, schizophrenia, and autism spectrum disorders (Ko, Choii, & Um, 2015; Pizzarelli & Cherubini, 2011; Sprekeler, 2017; Yizhar et al., 2011). The appropriate E/I ratio is dictated by different factors, with particular attention paid to different types of inhibitory neurons across brain regions (Isaacson & Scanziani, 2011; Keck, Hubener, & Bonhoeffer, 2017; Sprekeler, 2017).

There are two primary types of neurons in cortical brain areas: glutamatergic excitatory pyramidal neurons and γ‐aminobutyric acid (GABA)‐ergic interneurons. GABAergic interneurons are a diverse population of cells that can be classified by their morphology, electrophysiological and neurochemical features (Petilla Interneuron Nomenclature et al., 2008). The most common subtypes in neocortex are parvalbumin (PV), somatostatin (SST), and ionotropic serotonin receptor 5HT3a (5HT3aR)‐expressing interneurons (Rudy, Fishell, Lee, & Hjerling‐Leffler, 2011; Tremblay, Lee, & Rudy, 2016), each with particularly defined biophysical, postsynaptic targets and synaptic properties on excitatory cells. In fact, interneurons connect to other components of the neuronal network, both excitatory and inhibitory cells, following a detailed blueprint, that is, establishing a highly specific synaptic connectivity diagram between diverse neuronal types (Tremblay et al., 2016). Through inhibition, GABAergic interneurons shape the circuit activity controlling spike generation and frequency of neighboring pyramidal neurons (Roux & Buzsaki, 2015), setting feed‐forward excitation levels (Dichter & Ayala, 1987), and contributing to the fundamental synchronized oscillations found in brain networks.

Astrocytes constitute one of the major glial cell population in the mammalian brain (Herculano‐Houzel, Catania, Manger, & Kaas, 2015), with critical roles in homeostatic functions (Mederos, González‐Arias, & Perea, 2018). Astrocytes also encompass a large heterogeneous glial cell type (Matyash & Kettenmann, 2010; Zhang & Barres, 2010), including protoplasmic astrocytes, fibrous astrocytes, Müller cells, Bergman glia, perivascular glia, and velate astrocytes, each with particular molecular profiles (Farmer & Murai, 2017; Matyash & Kettenmann, 2010; Morel et al., 2017; Wu, Pan, Zuo, Li, & Hong, 2017; Zhang et al., 2016). Accumulated evidence has revealed the tight relationship between astrocytes, neurons, and synapses in the nervous system (Allen & Eroglu, 2017; Araque, Parpura, Sanzgiri, & Haydon, 1999; Gaudet & Fonken, 2018). In particular, astrocytes are recognized as key factors involved in synapse maturation, remodeling, and transmission that finally regulate synaptic plasticity (Araque et al., 2014; Eroglu & Barres, 2010; Rusakov, 2015). These actions can be achieved, among other mechanisms, by the release of active substances from astrocytes (so‐called gliotransmitters) such as glutamate, ATP and D‐serine, which activate pre‐ and postsynaptic receptors in neurons (Angulo, Kozlov, Charpak, & Audinat, 2004; Beattie et al., 2002; Chen et al., 2013; Covelo & Araque, 2018; Di Castro et al., 2011; Gomez‐Gonzalo et al., 2015; Henneberger, Papouin, Oliet, & Rusakov, 2010; Jourdain et al., 2007; Martin, Bajo‐Graneras, Moratalla, Perea, & Araque, 2015; Mederos et al., 2019; Perea & Araque, 2007; Petrelli et al., 2018; Shigetomi, Bowser, Sofroniew, & Khakh, 2008). Recent reports have revealed the contribution of astrocytes to modulation of different brain rhythms (Lee et al., 2014; Perea et al., 2016; Poskanzer & Yuste, 2016), forming the idea that astrocytes can participate in the complex operation modes of cortical networks. Remarkably, most of the current knowledge about astrocytic impact on synaptic transmission and plasticity comes from studies focused on excitatory synapses and excitatory pyramidal cells, leaving inhibitory synapses and GABAergic interneurons less explored (Lia, Zonta, Requie, & Carmignoto, 2018). This review will briefly highlight data focused on GABAergic inhibition and astrocytes, trying to increase our understanding about the astrocytic role in E/I balance and its impact on GABAergic‐driven operation modes of cortical networks.

## 2 ASTROCYTES SENSE GABAERGIC SIGNALING

When considering the relationship and consequences of astrocyte‐interneuron activity, some aspects must be considered: How do astrocytes sense GABA? Is interneuron to astrocyte signaling cell type‐specific? By what mechanisms do astrocytes contribute to the E/I balance and coordinated brain activity?

Along with a variety of membrane receptors and transporters for different neurotransmitters, astrocytes express GABA receptors and GABA transporters (GATs), specifically GAT‐1 and GAT‐3 (Boisvert, Erikson, Shokhirev, & Allen, 2018; Doengi et al., 2009; Ribak, Tong, & Brecha, 1996). GAT‐3 is the most abundant in astrocyte processes facing synapses and neuronal bodies (Boddum et al., 2016). GABA uptake via GAT‐1 and GAT‐3 activity influences GABAAR‐mediated inhibitory transmission (Moldavan, Cravetchi, & Allen, 2017; Song et al., 2013); but additionally, GAT‐3 activation stimulates the release of ATP/adenosine from hippocampal astrocytes, which contributes to downregulate excitatory transmission via activation of presynaptic adenosine receptors (A1Rs) (Boddum et al., 2016), and enhances inhibitory transmission via activation of postsynaptic A1Rs (Matos et al., 2018). This downregulation of excitatory transmission via GABAergic activation contributes to the hippocampal heterosynaptic depression phenomena (Boddum et al., 2016; Serrano, Haddjeri, Lacaille, & Robitaille, 2006). In thalamic astrocytes, GABA uptake via GAT‐1 contributes to reduce GABA spillover resulting from synaptic activity, and GAT‐3 activity regulates the concentration of GABA in the extrasynaptic space, regulating the magnitude of tonic inhibition (Beenhakker & Huguenard, 2010). It is known that several signals modulate the intracellular Ca^2+^ level within astrocytes, which prompts diverse cellular downstream mechanisms (Bazargani & Attwell, 2016; Rusakov, 2015). Thus, it is relevant that GAT‐3 activation in cerebellar (Doengi et al., 2009) and hippocampal astrocytes (Matos et al., 2018) enhances intracellular Ca^2+^ signaling. On the other hand, specific manipulation to downregulate Ca^2+^ signaling in striatal astrocytes has been linked to an increase in GAT‐3 expression in astrocytic membranes (Yu et al., 2018). Such enhancement of GAT‐3 activity was derived from a boost in GABA uptake, resulting in a reduction of tonic inhibition and exacerbated neuronal excitability, causing abnormal repetitive behavior phenotypes in mice (Yu et al., 2018). Then, these data indicate that GATs not only remove GABA from the extracellular space, but also contribute to modulate astrocytic intracellular signaling, showing the tight relationship between GABAergic interneurons and astrocytes.

Additionally, astrocytes express ionotropic GABA receptors (GABAARs) (MacVicar, Tse, Crichton, & Kettenmann, 1989; Muller et al., 1994) and metabotropic receptors (GABABRs) at the level of the soma, in the processes surrounding synapses and at the astrocyte endfeet in contact with blood vessels (Blomqvist & Broman, 1988; Lee et al., 2010; Martinez‐Rodriguez et al., 1993). While activation of GABAARs leads to hyperpolarization of neuronal membranes, it engages Cl^−^‐mediated astrocytic depolarizing currents in both cultured astrocytes and brain slices (Egawa, Yamada, Furukawa, Yanagawa, & Fukuda, 2013; Meier, Kafitz, & Rose, 2008; Muller et al., 1994), triggering astrocytic Ca^2+^ signaling via voltage‐sensitive Ca^2+^ channels (VOCCs) (Letellier et al., 2016; Meier et al., 2008; Tippens et al., 2008) upon membrane depolarization (Meier et al., 2008; Nilsson, Eriksson, Ronnback, & Hansson, 1993). An intense GABAergic activity induces Cl^−^ efflux from astrocytes that can regulate the driving force for neuronal GABAergic transmission by modulating extracellular Cl^−^ concentrations (Egawa et al., 2013), maintaining them at optimal levels during high interneuron firing.

GABABRs activation also induces Ca^2+^ signaling in astrocytes, in this case involving G_i/o_ proteins and Ca^2+^ release from intracellular stores (Mariotti, Losi, Sessolo, Marcon, & Carmignoto, 2016; Perea et al., 2016), including IP3R2‐sensitive sources (Mariotti et al., 2016; Perea et al., 2016; Sharp et al., 1999). Once Ca^2+^ signaling is engaged, GABA‐activated astrocytes can affect neuronal activity by releasing different gliotransmitters (glutamate, ATP, adenosine). A pioneer study in 1998 focusing on astrocytes and inhibitory signaling reported an enhancement of inhibitory transmission onto pyramidal cells via glutamate release from astrocytes and activation of ionotropic AMPA/NMDA glutamate receptors (Kang, Jiang, Goldman, & Nedergaard, 1998). Later, it was found that glutamate released from astrocytes also activated kainate receptors (Liu, Xu, Arcuino, Kang, & Nedergaard, 2004) and presynaptic II/III mGluRs at the inhibitory terminals (Liu, Xu, Kang, & Nedergaard, 2004), which inhibited transmitter release onto hippocampal interneurons. Alternatively, GABABR activation in hippocampal astrocytes also triggers the release of ATP/Adenosine (Ado) which causes a reduction in glutamate release at more distal synapses through activation of presynaptic A1Rs at glutamatergic terminals, resulting in short‐ or long‐term depression of hippocampal circuits such as heterosynaptic depression (Andersson, Blomstrand, & Hanse, 2007; Chen et al., 2013; Serrano et al., 2006). Overall, these results converge on the idea that by GABAergic activation, astrocytes can both spatially and temporally amplify interneuron actions to cover a large population of synapses, contributing to the inhibitory control of pyramidal networks.

## 3 INTERNEURON‐ASTROCYTE SIGNALING: TIMING MATTERS

The firing of GABAergic neurons and a tight regulation of pyramidal cell excitability is crucial for proper network activity (Tremblay et al., 2016); since astrocytes sense GABAergic activity, might they take part in such orchestrated signaling?

In order to control the firing rate of GABAergic interneurons and evaluate its impact on astrocyte networks and synapses, selective manipulation methods are used to stimulate interneurons. Experimental approaches using patch‐clamp recordings (Daw, Tricoire, Erdelyi, Szabo, & McBain, 2009; Yao et al., 2016) and optical stimulation (channelrhodopsin‐2; ChR2) (Adesnik, Bruns, Taniguchi, Huang, & Scanziani, 2012; Mariotti et al., 2018; Roux, Stark, Sjulson, & Buzsaki, 2014) allow to monitor the actions of a single interneuron or a particular population of cell type within the entire network. Thus, it has been found that GABA released during sparse low interneuron activity, that is, single action potentials or low firing rate during short periods by direct depolarization or optogenetic stimulation, does not stimulate Ca^2+^ signaling at the soma or unresolved domains in hippocampal and cortical astrocytes (Deemyad, Luthi, & Spruston, 2018; Perea et al., 2016; Rozsa et al., 2017), even though it is able to reach the astrocyte membranes inducing inward currents (Rozsa et al., 2017). However, interneuron‐firing rates above 5 Hz or at lower rate over long periods did stimulate intracellular somatic and local Ca^2+^ events in hippocampal astrocytes mediated by GABABRs and GAT‐3 (Covelo & Araque, 2018; Deemyad et al., 2018; Matos et al., 2018; Perea et al., 2016). In this scenario, whether astrocytes are recruited by enhanced or low but sustained GABAergic signaling, they would impact on hippocampal synaptic activity with different consequences. Indeed, interneuron firing rate at >40 Hz (gamma oscillations) evoked a midterm potentiation in subsets of excitatory CA3‐CA1 synapses (Perea et al., 2016). Such synaptic enhancement relied on group I mGluRs stimulation at presynaptic terminals by glutamate released from astrocytes (Perea et al., 2016). Hence, astrocytes transformed inhibitory (GABA) into excitatory (glutamate) signals, switching the output of interneuron activity in a subset of synapses (Covelo & Araque, 2018; Perea et al., 2016). With these actions, astrocytes are competent to expand the computation capabilities of particular CA1 neurons and the GABAergic functions into excitatory local networks. The magnitude of GABAergic potentiation of excitation showed a positive correlation with the firing rate of interneurons indicating the existence of a threshold for GABAergic tone to engage astrocyte‐driven excitatory synaptic responses (Perea et al., 2016). If this is the case, it would indicate that astrocytes can act as a high‐pass filter for GABAergic activity, favoring excitation over inhibition for particular CA3‐CA1 synapses.

Additionally, hippocampal astrocytes recruited by GABAergic activity can also release ATP/Ado (Andersson et al., 2007; Matos et al., 2018; Serrano et al., 2006). Hence, the same hippocampal circuit can experience potentiation and depression of excitatory synapses by GABAergic activation of astrocytes (Chen et al., 2013; Perea et al., 2016; Serrano et al., 2006), though the mechanisms motivating this dual effect are unclear. A recent study has investigated this issue at the hippocampal single synapse level (Covelo & Araque, 2018), finding that single GABAergic interneurons firing over 20 Hz recruited Ca^2+^ signaling in somatic compartments of nearby astrocytes, evoking both glutamate and ATP/Ado release. Interneuron‐astrocyte signaling induced a biphasic synaptic modulation of excitatory transmission, that is, transient potentiation followed by a longer‐lasting depression of excitation (Covelo & Araque, 2018). Indeed, sustained periods of GABAergic activity (90 s) over 10 Hz were sufficient to induce astrocyte‐driven glutamatergic‐mediated synaptic modulation, whereas shorter times (30 s) but higher interneuron firing rates (over 20 Hz) were required to evoke both glutamatergic and adenosine astrocyte‐mediated synaptic potentiation and depression, respectively (Covelo & Araque, 2018). These data indicate that astrocytes can decode the frequency and duration of interneuron activity by releasing different gliotransmitters and evoking a biphasic synaptic modulation of CA3‐CA1 synapses (Covelo & Araque, 2018). However, other studies in hippocampus and somatosensory cortex found that shorter durations (5 or 10 s) or different frequencies (2 or 40 Hz) of GABAergic activity were sufficient to engage astrocytic Ca^2+^ signaling (Deemyad et al., 2018; Mariotti et al., 2018; Matos et al., 2018; Perea et al., 2016) and synaptic‐derived modulation (Deemyad et al., 2018; Matos et al., 2018; Perea et al., 2016). Therefore, the broad spectrum of GABAergic actions on astrocytic physiology needs to be clarified, such as whether GABAergic‐astrocyte signaling express particular features in different brain areas, and its dependence on the interneuron firing rate, that is, duration and frequency. Considering that GABAergic‐interneurons operates at different frequency bands, such as theta (4–8 Hz) and gamma oscillations (>30 Hz) (Amilhon et al., 2015; Cardin, 2018), it could be expected that astrocytes were recruited by diverse interneuron activities, and by releasing glutamate and/or ATP/Ado they would participate from these network oscillations.

Overall, these data suggest that astrocytes contribute to categorize the synaptic weight received by pyramidal cells, favoring the excitation or inhibition force that finally drives the E/I ratio at the circuit level.

It is worth mention that single action potentials from glutamatergic axons have been found to engage localized Ca^2+^ signals in hippocampal astrocytes, using more controlled imaging of focal planes (Di Castro et al., 2011; Panatier et al., 2011) and three‐dimensional (3D) Ca^2+^ imaging in brain slices (Bindocci et al., 2017); however, somatic Ca^2+^ events were absent. The use of more powerful scanners has revealed higher frequency of local events in astrocyte processes than previously thought, suggesting that standard Ca^2+^ imaging approaches underestimate intracellular Ca^2+^ signaling in astrocytes, both in vitro and in vivo (Bindocci et al., 2017). Therefore, although current data supports the existence of a relative threshold in neuronal activity to stimulate astrocyte‐driven synaptic responses, further studies combining 3D Ca^2+^ imaging in astrocytes with optogenetics for specific interneuron cell‐type activation would reveal more sophisticated relationships between selective GABAergic axons and astrocytic processes, possibly uncovering novel functional signaling pathways in neural circuits.

## 4 INTERNEURON‐ASTROCYTE SIGNALING: GABAERGIC CELL‐TYPE MATTERS

GABAergic interneurons are highly heterogeneous, showing specific sets of cellular properties (Tremblay et al., 2016). Whether GABAergic‐astrocyte signaling shows differential features based on diverse interneuron cell types has been recently explored, taking advantage of molecular tools and transgenic mice to label specific GABAergic interneurons (Covelo & Araque, 2018; Mariotti et al., 2018; Matos et al., 2018; Perea et al., 2016). Indeed, it has been described that astrocyte Ca^2+^ responses to optical PV and SST interneuron activation show selective dynamics in the somatosensory cortex in vivo (Mariotti et al., 2018). The exceptional properties of SST‐driven astrocyte signaling, with high sensitivity to synaptic GABA and enhanced Ca^2+^ signaling compared to PV interneurons, were mediated by the release of neuropeptide somatostatin. SST (but not PV) cells co‐released neuropeptide somatostatin with GABA after intense activation (Katona et al., 2014; Mariotti et al., 2018). In addition, astrocytes displayed differential Ca^2+^ plasticity after successive episodes of PV and SST interneuron activity (Mariotti et al., 2018). Astrocyte Ca^2+^ signaling was stimulated by a first episode of sustained PV interneuron activity (1 Hz, 30 s), but upon successive PV cell firing at similar rate, Ca^2+^ signaling at soma and proximal processes were reduced. In contrast, successive periods of SST activity at similar firing rate induced astrocytic Ca^2+^ oscillation increases in those regions (Mariotti et al., 2018). In this study, most of the astrocytes recorded from layer 2–3 of the somatosensory cortex could be recruited by a second episode of SST firing, suggesting that intense activity in the SST interneuron circuit drives a long‐lasting activity in the astrocyte network (Mariotti et al., 2018). In this spirit, it has been reported that hippocampal astrocytes upregulate SST but not PV inhibitory synaptic transmission onto CA1 pyramidal cells via ATP/Ado release, which relies on activation of GAT‐3 in astrocytic membranes (Matos et al., 2018). However, it is unresolved whether the recorded astrocytes belong to different populations with specific preference for PV and SST GABAergic signaling, or there is a unique astrocyte population in the hippocampus that responds differently to both interneurons based on intrinsic cellular properties.

Additionally, ChR2‐activated astrocytes in the visual cortex can signal PV and SST interneurons via glutamate release, increasing the excitatory drive onto these cells, which results in a potentiation of the inhibitory synaptic transmission onto pyramidal cells (Perea, Yang, Boyden, & Sur, 2014). However, in vivo experiments showed that selective ChR2 stimulation of astrocytes boosted firing rate of PV interneurons, while pyramidal cells and SST interneurons showed either a decrease or a potentiation of spiking rate. Thus, astrocyte‐induced modulation of firing rates altered the E/I balance of the microcircuit, impacting the visual response properties of these neurons including orientation selectivity (Perea et al., 2014), one of the basic visual features coded by primary visual neurons (Lee et al., 2012). While orientation selectivity was bi‐directionally modulated in SST interneurons by astrocyte activity, PV interneurons showed a robust reduction in their response after astrocyte activation (Perea et al., 2014). GABAergic interneuron‐astrocyte signaling has been also described for cholecystokinin (CCK) interneurons (Crosby et al., 2018; Tan et al., 2017). Selective stimulation of hippocampal astrocytes with ChR2 increased the firing rate of CCK but not PV interneurons via ATP/Ado release, concomitantly reducing the spiking rate of CA1 pyramidal neurons (Tan et al., 2017). Such selective modulation had an impact on gamma oscillations induced by kainate ex vivo, reducing the power of gamma band by direct inhibition of pyramidal neurons (Armstrong & Soltesz, 2012; Tan et al., 2017). Altogether, these data indicate that cell type‐specificity of interneuron‐astrocyte signaling contributes to the homeostatic regulation of inhibitory inputs in dendritic and somatic regions of pyramidal neurons (Allene, Lourenco, & Bacci, 2015; Petilla Interneuron Nomenclature et al., 2008); and suggest that astrocytes could be involved in multiple aspects of information coding triggered by these GABAergic cells.

Other GABAergic interneurons that release different neuropeptides in addition to GABA such as neuropeptide Y (NPY), vasointestinal polypeptide (VIP), enkephalins and neurokinin B (van den Pol, 2012) might also show interneuron‐astrocyte specificity. Considering that astrocytes express receptors for these neuropeptides (Marin et al., 1991; Muller, Heinemann, & Berlin, 1997; van den Pol, 2012), it is possible that neuropeptide release by GABAergic interneurons enhances their recruitment of the neighboring astrocyte network, which may contribute to the particular actions governed by these interneurons. Recently, the existence of NPY‐astrocyte signaling was reported in hippocampal slices, where astrocyte networks contributed to NPY excitability and the particular firing properties of NPY cells (Deemyad et al., 2018). Whether this modulation is a particular feature of NPY cells or rather is a common response shown by other GABAergic cells needs to be resolved. Overall, current data highlights the importance of the context, in terms of brain area and local networks, for the specificity of interneuron‐astrocyte signaling.

It is important to note that not all inhibitory neurons participate equally in the E/I balance. Indeed, by changing the strength of the synaptic connection made by a single PV neuron to its target cell, a reduced inhibition could be achieved (Xue, Atallah, & Scanziani, 2014), whereas enhancing cellular activity granted a selective increase of PV‐mediated inhibition. In contrast, such manipulations in SST interneurons did not significantly impact SST‐mediated inhibition. Therefore, it seems that control of E/I ratio is primarily achieved by adjusting PV inhibition according to the cell's level of activity (Xue et al., 2014). In order to advance our knowledge of how brain networks finely coordinate the activity of different cell types, it is important to consider that astrocytes, with their ability to spatially and temporally expand the inhibitory effects and transform inhibitory signals into excitatory messages, contribute to dynamically shape the information flow through local circuits.

## 5 ASTROCYTES MEDIATE TONIC INHIBITION: EFFICIENT E/I BALANCE

In addition to the phasic, short‐lasting inhibition mediated by GABAARs, there are also long‐lasting forms of inhibition (i.e., tonic inhibition) that can be mediated by asynchronous or spontaneous vesicular release of GABA, and driven by high‐affinity, slowly desensitizing extrasynaptic GABAARs which sense low concentrations of extracellular GABA (Glykys & Mody, 2007; Wu et al., 2013). Due to its sustained effect, tonic inhibition dominates in controlling cell excitability over phasic inhibition (Cope et al., 2009; Farrant & Nusser, 2005), and it has been related to relevant physiological dysfunctions such as absence seizures, Alzheimer's disease and Huntington's disease (HD) (Cope et al., 2009; Jo et al., 2014; Wojtowicz, Dvorzhak, Semtner, & Grantyn, 2013). Astrocytes have been revealed as one of the main sources of extrasynaptic GABA that accounts for tonic inhibition (for a review see [Yoon & Lee, 2014]). Remarkably, astrocytes can synthesize GABA from the polyamine putrescine using an alternative pathway implying monoamine oxidase B (MAOB) (Heja et al., 2012; Yoon et al., 2014). Alterations in MAOB activity impaired tonic inhibition via GAT‐ or Bestrophin‐1 (Best1)‐dependent GABA release (Heja et al., 2012; Yoon et al., 2014). MAOB activity is also related to the molecular mechanisms used by astrocytes to convert network excitation into tonic inhibition. Thus, under intense glutamatergic neuronal excitation, glutamate is taken up by astrocytes and activation of glutamate transporters stimulates the putrescine‐GABA metabolic pathway, which provides the glial GABA that contributes to tonic inhibition of neurons (Heja et al., 2012). Such opponent effects provide a modulatory negative feedback, by which inhibition increases in line with higher activity in hippocampal networks. In vitro, this differential action of astrocytic glutamate and GABA transporters can regulate neuronal excitation during recurrent seizure‐like events, and in vivo, can reduce the power of gamma oscillations (Heja et al., 2012).

Astrocytes can release GABA via the Best1 channel which tonically activates both GABAARs and GABABRs, contributing to depression of excitatory synaptic transmission in hippocampal (Lee et al., 2010) and cerebellar networks (Jo et al., 2014; Yoon et al., 2014; Yoon & Lee, 2014), controlling motor coordination (Woo et al., 2018). A relevant feature of Best1 is its significant permeability to Cl in conjunction with GABA and glutamate in astrocytes (Lee et al., 2010; Park et al., 2009). Thus, activation of Best1 channels in physiological conditions not only causes astrocytes to release GABA, enhancing the tonic inhibition, but also to release glutamate, which induces several actions on synaptic transmission (Araque et al., 2014). By the release of these two important transmitters at local circuits, astrocytes are able to regulate E/I balance. However, structural biology studies of crystalized Best1 channel have shown that due to the reduced diameter of the aperture in the structure of the channel (∼3 Å) (Vaisey, Miller, & Long, 2016), Best1 allowed small ions to pass, but remained essentially impermeable to larger molecules, such as glutamate or GABA (Kane Dickson, Pedi, & Long, 2014). Therefore, more cautious analysis has to be done in astrocytes to unequivocally ensure that Best1 channel can permeate GABA and glutamate from astrocytes under physiological and pathological brain states.

Astrocytic control of extracellular GABA concentration can also be achieved by modulating GAT activity. Indeed, glial GABA transporters face the extrasynaptic space instead of the synapse (Kinney & Spain, 2002), which favors activation of extrasynaptic GABAARs and the consequent boost of tonic inhibition on neurons. During intense periods of neuronal activity, cortical astrocytes negatively regulate GAT1/3 function via activation of purinergic‐ and Ca^2+^‐dependent signaling, reducing GAT‐mediated GABA uptake (Jacob, Vaz, Ribeiro, & Sebastiao, 2014), and contributing to set tonic inhibition levels. The ability of astrocytes to release GABA might be affected in pathological conditions such as HD, where astrocytes show a reduced activity via GAT‐3 that decreases extracellular GABA and contributes to the hyperexcitation of striatal circuits (Wojtowicz et al., 2013).

GABA release from astrocytes can also be achieved by phasic modes using vesicular‐dependent mechanisms (Wang, Sun, Hou, & Hamilton, 2013). Indeed, astrocytes from the olfactory bulb were able to discretely release GABA, evoking hyperpolarizing inhibitory currents in mitral and granule cells, but also to release glutamate which activated NMDA receptors in granule cells (Kozlov, Angulo, Audinat, & Charpak, 2006). These both currents can synchronously occur in adjacent neurons, with a significant impact on the global network, that is, blocking neuronal firing for several hundred milliseconds (astrocytic GABA) or increasing the excitability of a particular set of neurons (astrocytic glutamate) (Kozlov et al., 2006). Although the way in which particular network activity controls astrocytic release of GABA in tonic or phasic modes is still undefined, current data indicates that glial cells possess a specialized synthesis pathway and distinct release machinery for GABA to modulate neuronal excitability via tonic inhibition, which impacts brain function and behavior (Woo et al., 2018).

Sharp inhibition in each principal neuron is crucial for the proper function of a microcircuit, and different inhibitory inputs must be coordinated in a stimulus‐dependent manner (Ko et al., 2015). In addition to rapid inhibitory actions, interneurons rule long‐range and long‐term forms of synaptic plasticity, such as lateral inhibition, feed‐forward inhibition, and top‐down control of sensory processing (Kepecs & Fishell, 2014). In this spirit, the potential astrocytic impacts on inhibitory synaptic transmission would have important functional consequences at global network level. Actually, this is the case found for different brain areas (Bowser & Khakh, 2004; Kang et al., 1998; Lalo et al., 2014; Perea et al., 2014), such as the amygdala. In this brain region, the endocannabinoid‐specific activation or specific manipulation of astrocytes with Designer Receptor Exclusively Activated by Designer Drugs (DREADD) enhanced inhibitory synapses from the lateral subdivision to the central amygdala neurons. The boosted inhibition was mediated by ATP/Ado release from astrocytes, and resultant activation of neuronal adenosine A2_A_ receptor (Martin‐Fernandez et al., 2017). In contrast, under the same type of stimuli, astrocytes from the central amygdala depressed excitatory inputs coming from the basolateral amygdala via A1R activation. The net consequence of such dual activities of astrocytes was a reduction in the firing rate of centromedial neurons in vivo (Martin‐Fernandez et al., 2017). Because basolateral nuclei are primarily involved in pondering the emotional content of sensory inputs, and centromedial nuclei are responsible for regulating levels of attention and arousal (LeDoux, 2000; Mosher, Zimmerman, & Gothard, 2010), the reduced firing rate of centromedial neurons by astrocyte stimulation may influence related behavioral outputs, such as fear expression (Martin‐Fernandez et al., 2017).

Additionally, in the hypothalamus astrocytes have been found to be involved in feeding behaviors (Argente‐Arizon, Freire‐Regatillo, Argente, & Chowen, 2015; Kim et al., 2014). In dorsomedial hypothalamus, CCK peptide, which acts a satiety signal and is enriched in this brain area (Chen, Scott, Zhao, Moran, & Bi, 2008), also stimulates Ca^2+^ signals in astrocytes (Crosby et al., 2018; Muller et al., 1997), triggering ATP release (Crosby et al., 2018). Interestingly, Ca^2+^ signals were also dependent of mGluR5 activation, indicating that concerted activity of glutamatergic fibers and CCK could engage intracellular Ca^2+^ into astrocytes (Crosby et al., 2018). After astrocytic enrollment by glutamate and CCK released by an intense neuronal activity (high frequency stimulation protocols), a shift in synaptic plasticity was found in orexin neurons, from long‐term synaptic depression to long‐term potentiation of GABAergic transmission (Crosby et al., 2018). Microinjections of CCK into the dorsomedial hypothalamus were able to generate long‐lasting inhibition of food intake in rats (Chen et al., 2008); therefore, astrocytes, via CCK activation, can increase the weight of inhibitory inputs to orexin neurons contributing to reduce food intake (Clerc et al., 2007).

## 6 CONCLUDING REMARKS

The emerging picture indicates that astrocyte activity driven by inhibitory cells modulates both excitatory and inhibitory synapses. Cortical circuits, responsible for higher brain functions (working memory, cognitive flexibility, attention, social interaction and emotional regulation), show a complex cellular architecture, but only ~20–30% of these cells are interneurons (Markram et al., 2004), even though they play crucial roles in diversifying and increasing the computational power of cortical networks. Hence, it might be possible that astrocytes activated by GABAergic interneurons can participate in their inhibitory actions by covering large populations of synapses and adding an extended temporal window for neuronal coding.

**Figure 1:**
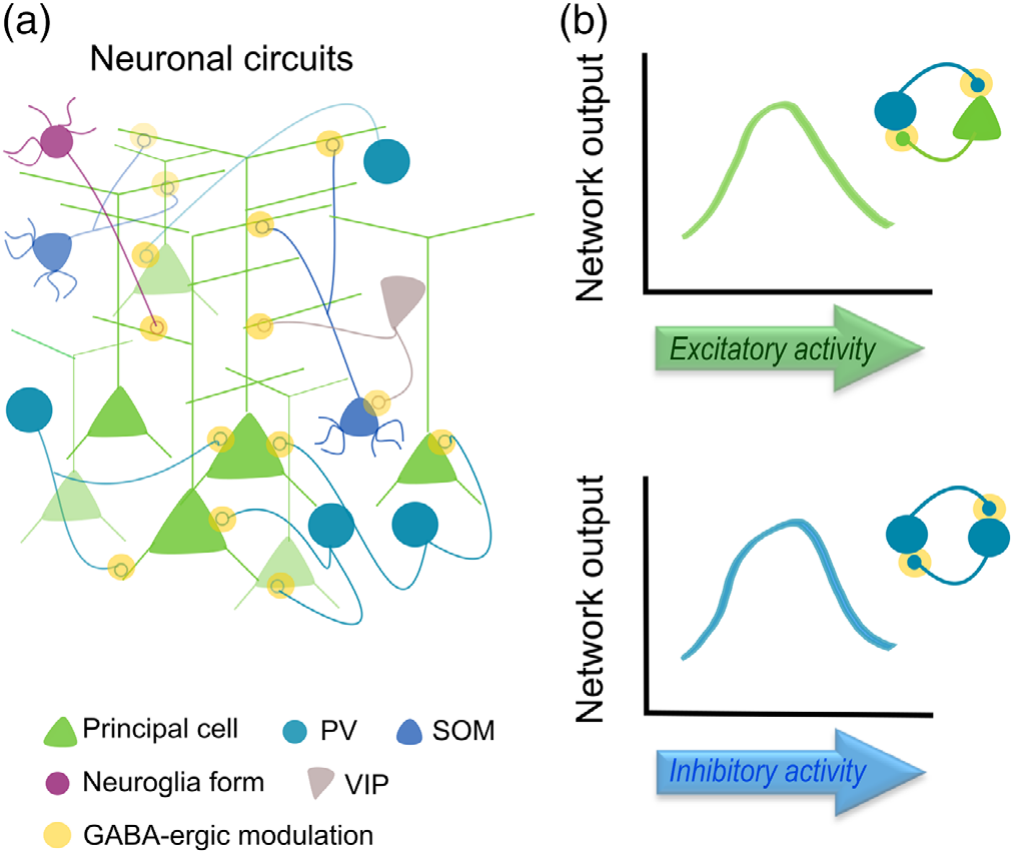
Schematic cortical circuit showing different neuron types interaction and their functional outputs. (a) Neuronal circuits based on interneuron‐principal cell connectivity. Yellow dots represent GABAergic point‐to‐point signaling. (b) GABAergic modulation of network activity responses driven by excitatory (top) and inhibitory inputs (bottom) in canonical neuronal circuits

**Figure 2:**
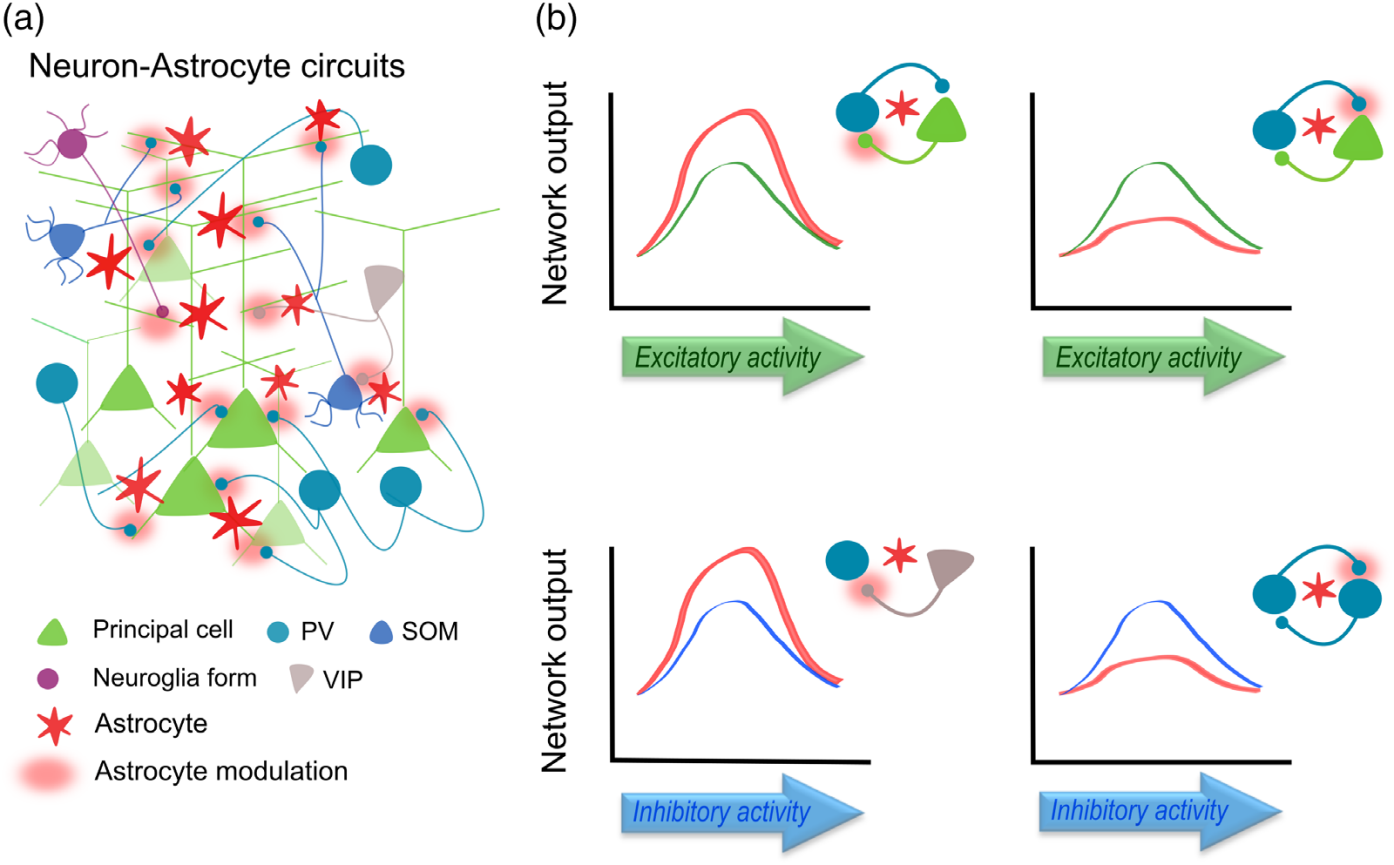
Schematic cortical circuit including astrocytes. (a) Astrocytes superimpose an additional layer of signaling to GABAergic synapses, which involves distant synapses and different time scales. (b) Astrocytes modulate both the excitatory (top) and inhibitory (bottom) drive onto principal cells and interneurons (red dots), enhancing or reducing synaptic activity and final network. Note that astrocyte impact on particular excitatory and inhibitory synapses contributes to increase the computation capabilities of cortical networks

Interneuron‐astrocyte signaling has particular features dictated by the specific GABAergic cell type and specific synapses, which enables astrocytes to actively regulate E/I balance using different mechanisms (GAT transporters, gliotransmission, etc.). As a result, such specific GABAergic‐astrocyte signaling would allow astrocytes to actively participate in diverse behaviors driven by particular interneuron subtypes. Experimental data and theoretical approaches have demonstrated that networks formed by purely excitatory connections provide little computational complexity; however, adding GABAergic interneuron signaling tightly regulates their excitation at a millisecond time scale by dynamically modulating the gain of excitatory neuronal outputs from simple action potential responsiveness (Buzsaki & Wang, 2012). Therefore, as well as interneurons, by way of their inhibitory actions, provide the necessary flexibility and independence to neighboring principal cells (Figure 1); astrocytes, by encoding temporal and spatial GABAergic signals, regulate the synaptic weight of excitatory and inhibitory cells, extending the computational power of local circuits (Figure 2). Considering the exquisite catalogue of interneuron subtypes, it is still unknown whether astrocytes show such specialization, that is, astrocytic subpopulations with preferential signaling for particular GABAergic interneurons, or even more, whether such interneuron‐astrocyte specialization can be regulated by the particular brain region they are located. Future studies combining molecular strategies to target select interneuron cell types with advanced imaging techniques (3D Ca^2+^ imaging), both in vitro and in vivo, will contribute to our understanding of the precise inhibitory synapse‐astrocyte organization at the molecular and functional level. Additionally, they will bring insights regarding the particular role of astrocytes in the currently considered interneuron‐driven neural phenomena; such as, feedback (“top‐down”) and feedforward inhibition (“bottom‐up”) of information flows in brain circuits, which allow to adjust weights of different inputs in a context‐dependent manner (Isaacson & Scanziani, 2011; Makino & Komiyama, 2015; Wyatte, Herd, Mingus, & O'Reilly, 2012). Therefore, further evidence of the power of this parallel signaling pathway controlling cortical E/I balance will expand our knowledge on higher functions of brain activity, and open the door to new therapeutic interventions against devastating neural disorders.

## CONFLICT OF INTEREST

The authors declare no competing financial interests.
